# Obstructed Breathing from Anæthesia

**Published:** 1890-02-01

**Authors:** 


### OBSTRUCTED BREATHING FROM ANÆSTHESIA.

In connection with the report of the Hyderabad Chloroform Commission, we mention that Dr. James Foulis, of Edinburgh, has recently reprinted his paper read before the Medico-Chirurgical Society of Edinburgh in March, 1889, "On the Only Way of Raising the Tongue and the Epiglottis, and of Stretching the Aryteno-Epiglottic Folds at One and the Same Time." Dr. Foulis states, from experiment, that it is the epiglottis which cuts off the supply of air. But if the base of the tongue and the hyoid bone can be drawn forwards, either by muscular action or by other means, it is impossible for the epiglottis to fall against the pharyngeal wall, or in any way obstruct the passage of air into the lungs during inspiratory efforts. One of the first well-marked effects of chloroform anæsthesia is relaxation of muscles. When a patient is lying on the back in a state of chloroform narcosis, those muscles which attach the tongue to the lower jaw, to the hyoid bone, and to the styloid process, become relaxed, and from this cause the tongue, with the hyoid bone moving on the thyreo-hyoid membrane as a hinge, has a tendency to fall backwards and to come in contact with the pharyngeal wall, in which position it, with the epiglottis, effectually prevents inspiration. Now when a patient is in danger of suffocation it has been the practice to drag the tongue upwards and forwards, and the Hyderabad Chloroform Commission also advise this. But Dr. Foulis states it only stretches the fibres of the tongue. "We may take it as a fact that dragging forcibly on the tip of the tongue with a pair of catch forceps cannot directly affect the hyoid bone or the epiglottis." The good effects of dragging on the tongue arises from the stretching of its fibres, so that the tongue, being drawn from the pharyngeal wall, allows the epiglottis to rise by its own elasticity. But there is always a danger of its acting as a valve during the inrush of air in inspiratory efforts. This is what undoubtedly occurs when dragging the tongue does not give relief. What we require is a simple means by which we can with certainty, under all conditions of apnœa, raise up the whole tongue and the epiglottis from contact with the posterior pharyngeal wall. It is not enough simply to make tense the elevator muscles of the hyoid bone, and to raise the epiglottis (as proposed by Howard) by bending the head back to the utmost, for there is danger in this method, and the Hyderabad Commission only say the head should be lowered. Neither is pushing or pulling forward the lower jaw sufficient, although according to the Hyderabad investigators this should be done. Bearing in mind the anatomical fact, that the base of the tongue is intimately connected with the hyoid bone and the epiglottis, it will be seen that if the base can be tilted or hooked forward the hyoid bone and the epiglottis will at the same time go forward. This can be done with the forefinger, but it is better and more conveniently accomplished with an instrument described by Dr. Foulis. Or one of the curved ends of the glosso-tilt should be passed down over the dorsum of the patient's tongue as far back as the base, and then the instrument should be pressed back in such a way that it acts as a lever. The first thing to be done is to cause the patient's head to fall well back over the end of the table, or two pillows may be placed below his shoulders. In the next place it is simple to insert the instrument between the tongue and the pharyngeal wall. The handle of a spoon will suffice. It is only necessary to insert such an instrument between the teeth and then push it onwards on the surface of the tongue till it comes in contact with the pharyngeal wall. If the handle of the spoon is now pressed back the whole tongue will be carried forward, and all parts lying in front of the handle of the spoon may be lifted forward with the greatest ease, allowing the ingress of air. Dr. Foulis observes: "There is no theory in all this. The method has been thoroughly put to the test, and it has never failed. It is applicable to every case where it is necessary to remove obstruction to breathing." The author gives various plates, illustrative of the anatomy of the parts and of the manner of using the glosso-tilt. The Hyderabad Chloroform Commission do not mention Dr. Foulis's method. Perhaps the members were not aware of what Dr. Foulis advocates. It would, however, have been satisfactory had the attention of the commission been directed to this matter.

